# Sources of Dietary Fiber Are Differently Associated with Prevalence of Depression

**DOI:** 10.3390/nu12092813

**Published:** 2020-09-14

**Authors:** Chong-Su Kim, Seohyeon Byeon, Dong-Mi Shin

**Affiliations:** 1 Department of Food and Nutrition, College of Human Ecology, Seoul National University, Seoul 08826, Korea; jskim11@snu.ac.kr (C.-S.K.); shmymy@snu.ac.kr (S.B.); 2 Research Institution of Human Ecology, Seoul National University, Seoul 08826, Korea

**Keywords:** dietary fiber, depression, gut–brain axis, KNHANES

## Abstract

Dietary fiber has been actively studied for its profound impacts on mental health by affecting the gut–brain axis communication. However, the association between dietary fiber intake and depression has been inconsistent, partly due to the lack of consideration of the fiber source. Therefore, this study aimed to examine the association between various sources of dietary fiber and depression in Korean adults through a nationwide cross-sectional study. The study population was a total of 2960 adults between 19 and 64 years of age who participated in the Korean National Health and Nutrition Examination Survey (KNHANES, 2012–2016). Dietary fiber intake from each fiber subtype (crude, cereal, vegetable, fruit, seaweed, and mushroom) was calculated using the Food Frequency Questionnaire (FFQ). Depression prevalence was assessed using a Patient Health Questionnaire (PHQ-9) and self-reported clinical diagnosis by a physician. We found that seaweed (odds ratio (OR) = 0.38; 95% confidence interval (CI): 0.20–0.72; *p* < 0.05) and mushroom fiber intake (OR = 0.18; 95% CI: 0.01–0.37; *p* < 0.05) were inversely associated with depressive symptoms assessed using the PHQ-9 parameters. Moreover, seaweed fiber intake was inversely associated with clinical depression diagnosed by a physician (OR = 0.45; 95% CI: 0.23–0.88; *p* < 0.05). This was the first study to find that higher intakes of seaweed and mushroom fiber were associated with a lower likelihood of depression in a representative cohort of Korean adults, indicating that the specific source of dietary fiber may be an important dietary factor in modulating depression.

## 1. Introduction

Depressive disorder is one of the most serious disabling public health problems worldwide with an extremely high prevalence. In 2015, 322 million people, or 4.4% of the global population, had depression, and the proportion was 4.1% in South Korea [1]. In addition, one of the major complications of depression is suicide [2], which caused 788,000 deaths in 2015, accounting for almost 1.5% of the total deaths [1]. However, traditional pharmacotherapies, including monoaminergic anti-depressants, selective serotonin reuptake inhibitors (SSRIs), and serotonin–norepinephrine reuptake inhibitors (SNRIs) [3], produce temporary remission in <50% of patients [4,5], and have a high adverse event rate [6]. Therefore, novel therapeutic or preventative approaches based on different mechanisms are urgently needed.

Emerging evidence shows that dietary patterns and specific dietary factors are linked to mental illness, including depression. For example, a traditional Western diet with a higher intake of processed food and/or fat and simple carbohydrates is associated with an increased risk for depression and anxiety [7]. The Mediterranean dietary pattern, which includes plenty of vegetables, fruits, and whole grains [7], is associated with fewer depressive symptoms [8,9]. In addition, several studies have suggested that specific dietary factors, such as ω-3 polyunsaturated fatty acids and dietary amino acids, have regulatory effects on depression [10,11]. It has been reported that a diet rich in tryptophan, a dietary amino acid, is implicated in stress-related mood disorders and affective changes by affecting serotonergic neurotransmission [12]. Collectively, dietary patterns and diets rich in specific nutrients have received increasing attention for their roles in regulating mental health.

Dietary fiber refers to the edible part of plants or analogous carbohydrates that are not digested and absorbed by humans. They are either partially or completely fermented in the large intestine [13]. Studies on dietary fiber have associated insufficient intake of it with Western diseases, such as diabetes, heart disease, or colorectal cancer, and diseases related to dysregulation of the immune system [14]. It has been suggested that the gut microbiota is a missing link in the association between low dietary fiber intake and those diseases since dietary fiber is a major modulator of the gut microbial composition [15,16,17]. Moreover, based on recent findings on the gut microbiota, it has been shown that gut microbes interact with the brain via the gut–brain axis, and thereby regulate brain functions including mood, behavior, and cognitive function [18,19]. These studies provide the potential implication that dietary fiber might have a regulatory role in mental diseases by affecting the gut–brain axis.

In fact, there has been growing interest in the association between dietary fiber, which modulates intestinal bacteria, and mental health, but the results of studies have been controversial. For example, fiber consumption showed an inverse association with depressive symptoms in a cross-sectional study of a US population [20] and a cohort study performed on American postmenopausal women [21]. However, there was no significant association in a cross-sectional study on an older Australian population [22]. In addition, a higher fiber intake from vegetables and fruits was associated with a lower risk of depressive symptoms. However, the total, cereal, soluble, and insoluble fiber consumption was not associated with the risk of depressive symptoms in a cross-sectional study performed on Japanese employees [23]. Overall, previous studies on the association between dietary fiber intake with depression showed inconsistent results depending on the population and fiber source. These results prompted us to assess the association between depression and the intake of dietary fiber derived from commonly consumed foods, particularly among the Korean population [24]. Therefore, we aimed to identify the association between depression and various dietary fiber sources (e.g., cereal, vegetables, fruits, seaweed, and mushrooms) in a Korean population via a nationwide cross-sectional study.

## 2. Materials and Methods

### 2.1. Design and Study Population

We used data from the Korean National Health and Nutrition Examination Survey (KNHANES V, KNHANES VI-1) conducted from 2012 to 2016 to perform a cross-sectional study on the Korean adult population. KNHANES is a health and nutrition survey of Koreans administered by the Korean Centers for Disease Control (KCDC) and Prevention and the Korean Ministry of Health and Welfare, and this study performed a complex, stratified, multistage, and probability-clustered survey on a representative sample [25].

Two separate analyses were conducted based on two different methods for evaluating the prevalence of depression. In the first study, depressive symptoms were determined using Patient Health Questionnaire-9 (PHQ-9) data, and their association with fiber intake from different sources was analyzed using the KNHANES 2016 data. A total of 2414 participants were included for the analysis after excluding participants with missing data. In the second study, the association between a clinical diagnosis of depression and fiber intake from different sources was analyzed using the KNHANES 2012–2015 data, and a total of 546 participants were included in the analysis after excluding participants with missing data.

### 2.2. Assessment of Dietary Fiber Intake

Diet assessments were performed using the food frequency questionnaire (FFQ). Total energy and fiber intake were calculated by multiplying the frequency of consumption by the nutrient composition for the portion size of each food item. Fiber intake from each food item was summed across all foods to obtain the total levels of fiber intake. Subgroups of fiber were categorized based on the food kingdom group, and fiber intake from each fiber subtype (crude, cereal, vegetable, fruit, seaweed, and mushroom) was calculated. Fiber intake was categorized into quartiles (Q1–Q4).

### 2.3. Evaluation of Severity and Prevalence of Depression

Depression was evaluated in two different ways. In the first analysis, PHQ-9, a depression screening tool with high sensitivity and specificity [26,27,28], was used to assess the severity of depressive symptoms. It consists of nine questions, each of which is scored from 0 (“not at all”) to 3 (“nearly every day”), resulting in a total score range of 0 to 27 [25]. Participants with total scores of ≥10 were classified into the moderate to severe depression group; those from 5 to <10, into the mild depression group; and those with <5, into the normal group. Participants with missing data were excluded. In the second analysis, the clinical diagnosis of depression by a physician was used to assess the prevalence of depression. The participants were classified into either the clinical depression group or the non-depression group based on their binary answer (yes/no) to the question: “Have you ever been diagnosed with depression by a physician?”

### 2.4. Statistical Analysis

For the comparison of continuous values across the depression groups, a *t*-test or analysis of variance (ANOVA) was used. For the comparison of categorical values across the groups, a chi-squared test or Fisher’s exact test was used. 

Based on the literature, we used the following variables as potential confounders: age, sex, body mass index (BMI), household income, educational level, smoking status, alcohol usage, physical activity, overall health status, and daily energy intake. Household income was categorized into four quartiles. Educational level was similarly categorized into four groups: elementary school or less, middle school, high school, and college or more. For smoking status, we defined a smoker as someone who ever smoked >100 cigarettes before and is currently using cigarettes. For alcohol usage, we classified the participants according to whether they consumed alcohol at least once a month for the last 12 months. For physical activity, we classified the participants as active or inactive according to whether they responded, “more than five times a week and >30 min each time” or not to the question “how many days and how much time do you spend on physical activity such as walking?” For the overall health status, the participants were classified into five groups according to their self-recognized overall health status as excellent, very good, good, fair, or poor. For the daily energy intake, FFQ data were used. 

We used multivariate logistic regression analysis to examine the associations between crude, cereal, vegetable, fruit, seaweed, and mushroom fiber intake and depression. We adjusted for age and sex as confounders in model 1, and further adjusted for BMI, household income, educational level, smoking status, alcohol usage, physical activity, overall health status, and daily energy intake as confounders in model 2. Two-tailed *p*-values of <0.05 were considered statistically significant. All statistical analyses were performed using SAS 9.4 (SAS Institute, Cary, NC, USA).

## 3. Results

### 3.1. General Characteristics of the Study Population

In the first study, we examined the association between the intake of different types of dietary fiber and PHQ-9 depressive symptoms. A total of 2414 participants were categorized into three groups depending on PHQ-9 depressive symptoms. The general characteristics of the participants in the PHQ-9 depressive symptom groups are shown in Table 1. The number (%) of participants in the normal, mild, and moderate to severe PHQ-9 depressive symptom groups were 1930 (79.95%), 360 (14.91%), and 124 (5.14%), respectively (Table 1). Age differed significantly between the groups (*p* < 0.01) and the participants with more severe depressive symptoms were more likely to be female and smokers (*p* < 0.0001). BMI, educational level, drinking status, and energy intake were not significantly different between the groups. However, physical activity, economic status, health status, and intakes of crude, cereal, vegetable, and fruit fiber were significantly different between the groups (*p* < 0.05). Notably, the moderate to severe depressive symptom group consumed significantly less seaweed and mushroom fiber (*p* < 0.05). 

In the second analysis, we evaluated the association between the intake of different types of dietary fiber and the prevalence of clinical depression diagnosed by a physician. A total of 546 participants were included in the analysis and the general characteristics of the participants are shown in Table A1. There were 198 (36.26%) participants diagnosed with clinical depression (Table A1). The individuals included in the clinical depression group showed significantly higher BMIs, lower economic status, and poor health status compared to those who had no clinical depression (*p* < 0.01). In addition, the subjects diagnosed with clinical depression had significantly lower energy intake, with lower intakes of crude, cereal, vegetable, seaweed, and mushroom fiber compared to those without clinical depression (*p* < 0.05).

### 3.2. Association between Dietary Fiber Intake and PHQ-9 Depressive Symptoms

Table 2 shows the odds ratios (OR) of PHQ-9 depressive symptoms depending on the intake of crude, cereal, vegetable, fruit, seaweed, and mushroom fiber. In univariate logistic regression analysis, cereal (unadjusted OR = 0.46; 95% CI: 0.27–0.78), vegetable (unadjusted OR = 0.66; 95% CI: 0.48–0.92), fruit (unadjusted OR = 0.30; 95% CI: 0.17–0.52), seaweed (unadjusted OR = 0.34; 95% CI: 0.20–0.60), and mushroom fiber (unadjusted OR = 0.21; 95% CI: 0.12–0.39) intake was inversely associated with PHQ-9 depressive symptoms among the participants with fiber intake in the fourth quartile (Q4) compared to those in the first quartile (Q1) group (*p* < 0.05). After adjusting for age and sex (model 1), cereal (adjusted OR = 0.48; 95% CI: 0.28–0.82), fruit (adjusted OR = 0.27; 95% CI: 0.15–0.47), seaweed (adjusted OR = 0.33; 95% CI: 0.19–0.58), and mushroom (adjusted OR = 0.20; 95% CI: 0.11–0.36) fiber intake remained significant among the participants in the Q4 group compared to those in the Q1 group (*p* < 0.05), but vegetable fiber intake was no longer significant. After adjusting for all covariates (model 2), the highest quartiles of seaweed (adjusted OR = 0.38; 95% CI: 0.20–0.72) and mushroom (adjusted OR = 0.18; 95% CI: 0.01–0.37) fiber intake were significantly associated with a lower prevalence of PHQ-9 depressive symptoms among the participants in the Q4 group compared to those in the Q1 group (*p* < 0.05).

### 3.3. Association between Dietary Fiber Intake and Clinical Depression Diagnosed by a Physician

The ORs of clinical depression based on the dietary fiber intake quartiles are shown in Table 3. No type of dietary fiber, including crude, cereal, vegetable, fruit, and mushroom fiber, was significantly associated with clinical depression in the unadjusted and adjusted models. However, the highest quartile of seaweed fiber intake (unadjusted OR = 0.39; 95% CI: 0.21–0.73; *p* < 0.05) was inversely associated with clinical depression in univariate logistic regression analysis and the statistical significance remained strong in the fully adjusted model (adjusted OR = 0.45; 95% CI: 0.23–0.88; *p* < 0.05). 

## 4. Discussion

In response to growing interest in the impact of the gut microbiota on mental health [18,29,30,31,32,33,34,35,36], research on the association between dietary fiber, which modulates commensal microbiota, and mental health, has expanded [37,38,39,40]. However, the results on the relationship between dietary fiber and depression have been inconsistent, partly due to the lack of consideration of fiber source [20,21,22,23]. Therefore, in the present study, we aimed to assess the association between depression and the intake of different sources of fiber, taking into account particular dietary habits of the Korean population, via a cross-sectional study using KNHANES data. The main findings of the present study were that seaweed and mushroom fiber intake was inversely associated with the severity of depressive symptoms; seaweed fiber intake was inversely associated with a clinical diagnosis of depression.

Dietary patterns have been reported to be associated with depression. For example, a Western dietary pattern characterized by processed food and/or high fat and simple carbohydrates [9] is associated with a high risk of depression [7]. On the other hand, the Mediterranean diet pattern, which includes plenty of vegetables, fruits, and whole grains [7], is associated with fewer depressive symptoms [8,9]. In addition, specific food items or nutrients, such as ultra-processed food [41,42] and sugar [43], are also known to be related to depressive symptoms. Based on these results, it seems that a diet low in fiber sources might be an important factor for greater risk of depression; therefore, it prompted us to examine if dietary fiber intake is a key component of the connection between diet and depression.

Dietary fiber is generally known to be abundant in vegetables, fruits, and cereals [24] and the positive impact of diets rich in vegetables and fruits on mental health has been documented regarding their antioxidative effects [44,45]. However, considering the impact of dietary fiber intake on the gut microbiota, recent studies have expanded the roles of dietary fiber in the regulation of mental health through the gut–brain axis. It is notable that the traditional Korean diet contains various sources of high fiber, including dietary fiber from seaweeds and mushrooms [24]. In fact, it has been reported that major sources of dietary fiber for Koreans are from seaweeds and mushrooms followed by vegetables and cereals [24]. However, the effect of dietary fiber consumption sourced from seaweeds or mushrooms on depression in the Korean population has not been studied. These particular dietary habits (or food choices) among the Korean population and their association with anti-depressive effects were of interest since the type of dietary fiber was shown to differentially affect the intestinal microbiota, which has crucial impacts on mental health. In the present study, we verified that seaweed and mushroom fiber intake was inversely associated with the severity of depressive symptoms. Moreover, we found that seaweed fiber intake was inversely associated with the clinical diagnosis of depression in Korean adults. Among the foods commonly consumed by Koreans, dried brown seaweed has the highest dietary fiber content, and sea tangle and laver, which are commonly consumed types of brown seaweed, have high dietary fiber contents of more than 30%. In addition, various types of mushrooms commonly consumed by Koreans are also good sources of dietary fiber [24]. 

In fact, it has been suggested that seaweed has the potential to be a neuroprotective agent due to its antioxidant and anti-inflammatory activities, which are mainly derived from the phenolic compounds found in seaweed [46]. In addition, a recent study showed that ulvan, a type of fiber from seaweed, had protective effects on the occurrence and development of Alzheimer’s disease by inhibiting Aβ aggregation in vitro [47]. However, the possible anti-depressive effects of seaweed and underlying mechanistic actions need to be investigated in a future study.

It is interesting that specific types of dietary fiber were found to be differently associated with depression among Koreans. This finding can be supported by emerging evidence that the fiber utilized as microbiota-accessible carbohydrates (MACs) differs between populations. In other words, the type of fiber that can exert beneficial effects on host physiology may differ by population. For example, genes for the algal polysaccharide porphyran in the microbiomes, which determine a carbohydrate qualified as a MAC, were present in Japanese people, but rarely found in North American and European individuals [48,49]. In addition, it has been suggested that host genotypes also play a role in defining MACs since the host’s genotype affects mucus structure [50] and the efficiency of digestion and the absorption of carbohydrates in the small intestine [51,52,53]. Thus, these findings may explain why specific sources of fiber were differently associated with depression in the present study. Moreover, in line with previous findings, our results support the notion that it is imperative to consider the source of dietary fiber in accordance with population characteristics to determine its beneficial effects on host health in future studies.

Several mechanisms explain how dietary fiber affects depression. Based on recent findings, dietary fiber affects microbiota diversity and the bacterial metabolites, such as short-chain fatty acids (SCFAs) [14], acetate, propionate, or butyrate [54]. These bacterial metabolites have been found to play diverse regulatory roles, including histone acetylation [55] and signaling through G protein-coupled receptors [56], and affect the host’s immune system [57]. Of note, it has been shown that gut microbiota decompose polysaccharides in seaweed into SCFAs [58]. In turn, it enhances the diversity of gut microbiota [59], impedes the growth of harmful microbes in the gut, and promotes the growth of beneficial microbes such as *Bifidobacterium* [58]. Additionally, mushrooms are rich in dietary fiber, such as α-glucan, β-glucan, chitin, or xylan [60], which are known to diversify gut microbiota, constrain the growth of pathogens, and improve the immune system [61]. Such changes in gut microbiota derived by the consumption of dietary fiber, the metabolites thereof, and the effects on the immune system are being increasingly shown to affect brain function and emotional behavior. For example, a deficiency in MAC consumption leads the gut microbiota to consume gut mucus [62], and mucus depletion results in a weaker immune system [63], which may increase inflammation, an important biological event that increases the risk of depression [64]. Furthermore, the effects of the gut microbiota on the immune system [65] can directly affect brain function by changing the circulating levels of pro-inflammatory and anti-inflammatory cytokines [64]. In addition, microbiota regulate serotonin concentrations, a neurotransmitter often targeted by anti-depressant drugs [66], in the brain of the host since gut microbiota affect the metabolism and availability of tryptophan, which is a precursor of serotonin [67]. Overall, there are complex mechanistic actions involved in the effects of dietary fiber on depression, which emphasizes the need for future studies investigating the underlying mechanisms regarding the results of the present study.

This study had some limitations. First, the results of the present study do not imply causality, as it was a cross-sectional study, and mechanistic actions underlying the potential effects of dietary fiber from seaweed and mushrooms on depression need to be investigated in future studies. Second, assessment of the severity and prevalence of depression was based on self-report, which might have caused recall bias. Moreover, other confounding factors may exist, although we adequately controlled for potential confounders based on previous studies.

## 5. Conclusions

Despite these limitations, to the best of our knowledge, this was the first study to verify significant associations between the intake of seaweed and mushroom fiber and a lower risk of depressive symptoms and clinical depression in a representative cohort of Korean adults. These results provide new insight that the source of dietary fiber may be an important factor regarding its beneficial effects on depression. Therefore, further study is warranted to confirm the effects of different types of dietary fiber on depression.

## Figures and Tables

**Table 1:** General characteristics (2016).

	PHQ-9 Depressive Symptoms			
	Normal	Mild	Moderate to Severe	*p*-Value
Participants	1930 (79.95)	360 (14.91)	124 (5.14)	
Age, y	42.82 (12.16)	40.57 (12.17)	42.1 (13.64)	0.0057
Sex				
Male	821 (42.54)	106 (29.44)	36 (29.03)	<0.0001
Female	1109 (57.46)	254 (70.56)	88 (70.97)	
BMI, kg/m^2^	23.78 (3.64)	23.48 (3.49)	23.97 (4.23)	0.2728
Educational level				
Elementary or less	128 (6.63)	31 (8.61)	27 (21.77)	0.2216
Junior high school	148 (7.67)	23 (6.39)	9 (7.26)	
High school	705 (36.53)	135 (37.50)	47 (37.90)	
College or more	949 (49.17)	171 (47.50)	41 (33.06)	
Household income				
Q1 (lowest)	134 (6.95)	39 (10.83)	31 (25)	<0.0001
Q2	454 (23.55)	90 (25)	37 (29.84)	
Q3	637 (33.04)	102 (28.33)	33 (26.61)	
Q4 (highest)	703 (36.46)	129 (35.83)	23 (18.55)	
Smoking status				
No	1591 (82.44)	282 (78.33)	79 (63.71)	<0.0001
Yes	339 (17.56)	78 (21.67)	45 (36.29)	
Drinking status				
No	943 (48.86)	165 (45.83)	60 (48.39)	0.5732
Yes	987 (51.14)	195 (54.17)	64 (51.61)	
Physical activity				
No	1172 (60.73)	240 (66.67)	86 (69.35)	0.0234
Yes	758 (39.27)	120 (33.33)	38 (30.65)	
Health status				
Excellent	111 (5.75)	5 (1.39)	0 (0)	<0.0001
Very good	607 (31.45)	54 (15)	7 (5.65)	
Good	1015 (52.59)	186 (51.67)	42 (33.87)	
Fair	180 (9.33)	105 (29.17)	49 (39.52)	
Poor	17 (0.88)	10 (2.78)	26 (20.97)	
Energy intake, kcal/d	1818.04 (614.15)	1840.10 (674.25)	1727.19 (706.29)	0.2207
Crude fiber, g/d	5.49 (2.23)	5.26 (2.35)	4.16 (2.08)	0.0013
Cereal fiber, g/d	4.83 (1.96)	4.77 (2.03)	4.16 (2.08)	0.0015
Vegetable fiber, g/d	5.58 (2.65)	5.20 (2.52)	4.61 (2.78)	<0.0001
Fruit fiber, g/d	2.3 (1.81)	2.14 (1.76)	1.77 (1.81)	0.0027
Seaweed fiber, g/d	0.76 (0.57)	0.77 (0.57)	0.6 (0.57)	0.0082
Mushroom fiber, g/d	0.09 (0.07)	0.10 (0.08)	0.06 (0.06)	<0.0001

BMI, body mass index; PHQ-9, Patient Health Questionnaire-9. All values are *n* (%) or mean (SD). Statistical significance based on ANOVA, chi-squared test, or Fisher’s exact test.

**Table 2:** Association between depressive symptoms and dietary fiber intake (KNHANES 2016).

	Crude			Model 1			Model 2		
	OR (95% CI)			OR (95% CI)			OR (95% CI)		
Depressive Symptom	Normal	Mild	Moderate to Severe	Normal	Mild	Moderate to Severe	Normal	Mild	Moderate to Severe
Crude fiber (g/d)									
Q1 (<3.72)	Reference	Reference	Reference	Reference	Reference	Reference	Reference	Reference	Reference
Q2 (3.72 to <5.16)	Reference	0.80 (0.58–1.08)	0.37 (0.22–0.62)	Reference	0.83 (0.60–1.13)	0.38 (0.222–0.634)	Reference	0.72 (0.49–1.08)	0.47 (0.22–0.97)
Q3 (5.16 to <6.85)	Reference	0.73 (0.54–1.01)	0.45 (0.28–0.74)	Reference	0.80 (0.59–1.11)	0.48 (0.29–0.78)	Reference	0.63 (0.36–1.10)	0.77 (0.29–2.07)
Q4 (≥6.85)	Reference	0.70 (0.51–0.96)	0.43 (0.26–0.71)	Reference	0.78 (0.56–1.07)	0.45 (0.27–0.74)	Reference	0.52 (0.21–1.29)	0.74 (0.15–3.65)
Cereal fiber (g/d)									
Q1 (<3.31)	Reference	Reference	Reference	Reference	Reference	Reference	Reference	Reference	Reference
Q2 (3.31 to <4.59)	Reference	0.93 (0.68–1.28)	0.63 (0.38–1.03)	Reference	0.94 (0.68–1.29)	0.64 (0.39–1.05)	Reference	0.80 (0.56–1.14)	0.63 (0.35–1.15)
Q3 (4.59 to <6.08)	Reference	0.99 (0.72–1.35)	0.73 (0.45–1.17)	Reference	1.04 (0.76–1.43)	0.77 (0.48–1.24)	Reference	0.85 (0.58–1.24)	0.80 (0.42–1.51)
Q4 (≥6.08)	Reference	0.84 (0.61–1.16)	0.46 (0.27–0.78) *	Reference	0.86 (0.62–1.19)	0.48 (0.28–0.82) *	Reference	0.63 (0.39–0.99)	0.51 (0.23–1.15)
Vegetable fiber (g/d)									
Q1 (<3.48)	Reference	Reference	Reference	Reference	Reference	Reference	Reference	Reference	Reference
Q2 (3.48 to <5.05)	Reference	0.86 (0.63–1.18)	0.46 (0.28–0.75)	Reference	0.89 (0.65–1.22)	0.47 (0.284–0.767)	Reference	0.81 (0.57–1.14)	0.50 (0.28–0.91)
Q3 (5.05 to <7.03)	Reference	0.90 (0.66–1.23)	0.42 (0.25–0.70)	Reference	1.00 (0.72–1.35)	0.45 (0.27–0.75)	Reference	0.47 (0.59–1.24)	0.63 (0.24–0.90)
Q4 (≥7.03)	Reference	0.66 (0.48–0.92) *	0.44 (0.27–0.72)	Reference	0.74 (0.53–1.03)	0.47 (0.29–0.78)	Reference	0.63 (0.40–1.01)	0.62 (0.28–1.36)
Fruit fiber (g/d)									
Q1 (<0.80)	Reference	Reference	Reference	Reference	Reference	Reference	Reference	Reference	Reference
Q2 (0.80 to <1.84)	Reference	0.90 (0.65–1.23)	0.44 (0.27–0.72)	Reference	0.85 (0.62–1.16)	0.41 (0.25–0.68)	Reference	0.97 (0.69–1.37)	0.73 (0.41–1.30)
Q3 (1.84 to <3.31)	Reference	0.96 (0.70–1.31)	0.30 (0.17–0.52) *	Reference	0.90 (0.65–1.23)	0.27 (0.15–0.47) *	Reference	1.18 (0.81–1.71)	0.63 (0.32–1.26)
Q4 (≥3.31)	Reference	0.78 (0.56–1.07)	0.51 (0.32–0.81)	Reference	0.72 (0.51–1.00)	0.44 (0.20–0.71)	Reference	1.09 (0.70–1.70)	1.52 (0.74–3.13)
Seaweed fiber (g/d)									
Q1 (<0.31)	Reference	Reference	Reference	Reference	Reference	Reference	Reference	Reference	Reference
Q2 (0.31 to <0.61)	Reference	1.29 (0.93–1.77)	0.41 (0.24–0.69)	Reference	1.23 (0.93–1.77)	0.39 (0.23–0.67)	Reference	1.36 (0.96–1.91)	0.48 (0.26–0.87)
Q3 (0.61 to <1.02)	Reference	0.97 (0.69–1.35)	0.64 (0.41–1.01)	Reference	0.99 (0.72–1.39)	0.63 (0.40–0.99)	Reference	1.04 (0.72–1.51)	0.80 (0.46–1.39)
Q4 (≥1.02)	Reference	1.22 (0.89–1.69)	0.34 (0.20–0.60) *	Reference	1.24 (0.89–1.71)	0.33 (0.19–0.58) *	Reference	1.22 (0.84–1.76)	0.38 (0.20–0.72) *
Mushroom fiber (g/d)									
Q1 (<0.03)	Reference	Reference	Reference	Reference	Reference	Reference	Reference	Reference	Reference
Q2 (0.03 to <0.08)	Reference	0.90 (0.64–1.25)	0.47 (0.30–0.74)	Reference	0.83 (0.59–1.16)	0.44 (0.276–0.690)	Reference	0.77 (0.54–1.10)	0.50 (0.26–0.76)
Q3 (0.08 to <0.14)	Reference	1.13 (0.83–1.56)	0.28 (0.16–0.48)	Reference	1.05 (0.76–1.45)	0.26 (0.15–0.45) *	Reference	1.02 (0.71–1.46)	0.25 (0.13–0.48)
Q4 (≥0.14)	Reference	1.04 (0.75–1.43)	0.21 (0.12–0.39) *	Reference	0.95 (0.69–1.32)	0.20 (0.11–0.36) *	Reference	0.90 (0.61–1.32)	0.18 (0.01–0.37) *

KNHANES, Korean National Health and Nutrition Examination Survey. * *p* < 0.05. Model 1: adjusted for age and sex; Model 2: adjusted for variables in model 1 plus economic status, education, smoking status, alcohol consumption, physical activity, subjective health status, BMI, and total energy intake.

**Table 3:** Association between clinical depression and dietary fiber intake (KNHANES 2012–2015).

	Crude	Model 1	Model 2
Clinical Depression	OR (95% CI)	OR (95% CI)	OR (95% CI)
Crude fiber (g/d)			
Q1 (<3.94)	Reference	Reference	Reference
Q2 (3.94 to <5.05)	0.52 (0.29–0.92)	0.50 (0.28–0.88)	0.49 (0.22–1.07)
Q3 (5.05 to <6.61)	0.67 (0.37–1.22)	0.66 (0.36–1.19)	0.76 (0.27–2.14)
Q4 (≥6.61)	0.56 (0.31–1.02)	0.55 (0.30–1.00)	0.54 (0.11–2.63)
Cereal fiber (g/d)			
Q1 (<3.20)	Reference	Reference	Reference
Q2 (3.20 to <4.82)	1.01 (0.57–1.79)	0.99 (0.56–1.75)	1.33 (0.66–2.67)
Q3 (4.82 to <6.60)	0.74 (0.41–1.36)	0.72 (0.39–1.34)	0.88 (0.42–1.83)
Q4 (≥6.60)	0.68 (0.37–1.26)	0.71 (0.39–1.30)	1.11 (0.47–2.61)
Vegetable fiber (g/d)			
Q1 (<3.48)	Reference	Reference	Reference
Q2 (3.48 to <4.86)	0.75 (0.42–1.33)	0.73 (0.41–1.30)	0.77 (0.38–1.56)
Q3 (4.86 to <6.45)	0.70 (0.39–1.26)	0.68 (0.38–1.24)	0.87 (0.39–1.92)
Q4 (≥6.45)	0.63 (0.35–1.15)	0.59 (0.32–1.09)	0.71 (0.29–1.75)
Fruit fiber (g/d)			
Q1 (<0.82)	Reference	Reference	Reference
Q2 (0.82 to <1.77)	0.85 (0.46–1.56)	0.88 (0.48–1.60)	1.33 (0.69–2.56)
Q3 (1.77 to <3.37)	0.63 (0.34–1.16)	0.64 (0.35–1.18)	0.97 (0.47–2.01)
Q4 (≥3.37)	0.75 (0.41–1.36)	0.77 (0.42–1.39)	1.29 (0.57–2.90)
Seaweed fiber (g/d)			
Q1 (<0.30)	Reference	Reference	Reference
Q2 (0.30 to <0.60)	0.65 (0.36–1.17)	0.64 (0.35–1.17)	0.74 (0.34–1.41)
Q3 (0.60 to <1.05)	0.56 (0.31–1.00)	0.58 (0.32–1.05)	0.76 (0.39–1.48)
Q4 (≥1.05)	0.39 (0.21–0.73) *	0.38 (0.21–0.71) *	0.45 (0.23–0.88) *
Mushroom fiber (g/d)			
Q1 (<0.02)	Reference	Reference	Reference
Q2 (0.02 to <0.05)	0.64 (0.35–1.15)	0.67 (0.37–1.22)	0.89 (0.45–1.74)
Q3 (0.05 to <0.10)	0.56 (0.31–0.99)	0.60 (0.34–1.09)	0.89 (0.47–1.73)
Q4 (≥0.10)	0.62 (0.34–1.13)	0.66 (0.36–1.22)	0.87 (0.44–1.72)

KNHANES, Korean National Health and Nutrition Examination Survey. * *p* < 0.05. Model 1: adjusted for age and sex; Model 2: adjusted for variables in model 1 plus economic status, education, smoking status, alcohol consumption, physical activity, subjective health status, BMI, and total energy intake.

## Appendix A

**Table A1:** General characteristics (2012–2015).

	Clinical Diagnosis of Depression		
	No	Yes	*p*-Value
Participants	348 (64.74)	198 (36.26)	
Age, y	45.95 (12.93)	47.12 (12.03)	0.2978
Sex			
Male	61 (17.53)	36 (18.18)	0.9074
Female	287 (82.47)	162 (81.82)	
BMI, kg/m^2^	23.39 (3.40)	24.24 (3.91)	0.0083
Educational level			
Elementary or less	67 (19.25)	47 (23.74)	0.2216
Junior high school	52 (14.94)	34 (17.17)	
High school	135 (38.79)	78 (39.39)	
College or more	94 (27.01)	39 (19.70)	
Household income			
Q1 (lowest)	43 (12.50)	63 (32.14)	<0.0001
Q2	99 (28.78)	57 (29.08)	
Q3	111 (32.27)	37 (18.88)	
Q4 (highest)	91 (26.45)	39 (19.90)	
Smoking status			
No	295 (84.77)	156 (78.79)	0.0794
Yes	53 (15.23)	42 (21.21)	
Drinking status			
No	211 (60.63)	122 (61.62)	0.8554
Yes	137 (39.37)	76 (38.38)	
Physical activity			
No	229 (65.99)	134 (67.68)	0.7066
Yes	118 (34.01)	64 (32.32)	
Health status			
Excellent	5 (1.44)	1 (0.51)	<0.0001
Very good	50 (14.37)	14 (7.07)	
Good	204 (58.62)	88 (44.44)	
Fair	81 (23.28)	72 (36.36)	
Poor	81 (23.28)	72 (36.36)	
Energy intake, kcal/d	1793.90 (638.50)	1605.80 (635.30)	0.0011
Crude fiber, g/d	5.62 (2.23)	5.03 (2.28)	0.0030
Cereal fiber, g/d	5.23 (2.24)	4.71 (2.34)	0.0103
Vegetable fiber, g/d	5.15 (1.95)	4.61 (2.02)	0.0021
Fruit fiber, g/d	2.53 (2.32)	2.16 (2.10)	0.0627
Seaweed fiber, g/d	0.83 (0.64)	0.70 (0.62)	0.0290
Mushroom fiber, g/d	0.08 (0.07)	0.06 (0.06)	0.0193

BMI, body mass index. All values are *n* (%) or mean (SD). Statistical significance based on *t*-test, chi-squared test, or Fisher’s exact test.
